# Geometrical and Electronic Analysis of Polyepoxysuccinic Acid (PESA) for Iron Sulfide Scale Inhibition in Oil Wells

**DOI:** 10.3390/polym14245433

**Published:** 2022-12-12

**Authors:** Patricia Magadia, Samah Abdulla, Elkhansa Elbashier, Ibnelwaleed A. Hussein, Mazen Khaled, Mohammed Saad

**Affiliations:** 1 Chemical Engineering Department, College of Engineering, Qatar University, Doha P.O. Box 2713, Qatar; 2 Gas Processing Center, College of Engineering, Qatar University, Doha P.O. Box 2713, Qatar; 3 Department of Chemistry and Earth Sciences, College of Arts and Sciences, Qatar University, Doha P.O. Box 2713, Qatar

**Keywords:** scale inhibition, density functional theory, polyepoxysuccinic acid (PESA), electronic structure, iron

## Abstract

Scale formation causes major losses in oil wells, related to production and equipment damages. Thus, it is important to develop effective materials to prevent scale formation and inhibit any additional formation. One known environmentally friendly material with promising performance for scale inhibition is polyepoxysuccinic acid (PESA). However, the performance and further development of any scale treatment chemical is highly affected by its electronic structure and behavior. Thus, this paper aims to obtain insights into the kinetics and thermodynamics of the chemical reactions during scale inhibition by investigating the geometrical and electronic structure of PESA. Density Functional Theory (B3LYP/6–31 g (d)-lanl2dz) was used to study the structure of PESA, considering different forms of PESA and their corresponding binding affinities and chemical behaviors. The scale is represented as Fe^II^ ions, and PESA is modeled as (n = 1, and 2). Three conditions of PESA were considered: the standard form, the form with a modified electron donating group (R- = CH_3_-), and ammonium salt of PESA (M^+^ = NH_4_^+^). The results showed that PESA has a high binding affinity to Fe^II^, comparable to known chelating agents, with the highest binding affinity for ammonium salt of PESA with the CH_3_- donating group (−1530 kJ/mol). The molecular orbitals (MO), electron affinity (EA), and charge analysis further explained the findings. The HOMO-LUMO gap and EA results revealed the high reactivity and thermodynamic stability of all forms of PESA. In addition, the ammonium salt form of PESA with the electron donating group performs better, as it has a greater overall negative charge in the compounds. Furthermore, the NH_4_^+^ cationic group tends to lower the value of the HOMO orbital, which increases the inhibition performance of PESA.

## 1. Introduction

Oilfield scales are solid deposits that build over time, obstructing fluid flow through pipelines and other equipment, resulting in severe production losses and equipment damage. The North Sea and Canada are examples of many areas encountering scale challenges. Due to scaling, North Sea well production fell from 30 k B/D to zero in just 24 h. As a result, oilfield scaling issues are expected to intensify and become more expensive in the future [1]. Oilfields have numerous scales, such as calcium carbonate, barium sulfate, sodium chloride, etc. [2]. However, one of the troublesome scales in oil and gas wells is the formation of iron sulfide [3]. The combination of iron and hydrogen sulfide in sour oil and gas production wells causes the iron sulfide scale to develop. These forms of iron sulfides include pyrrhotite, marcasite, mackinawite, and pyrite [4].

Scale inhibition methods must be rapid, non-destructive, and successful in avoiding re-precipitation. Knowing the kind and quantity of scale-forming ions is essential, as scale recurrence can be accelerated by using the wrong inhibition procedures. In oil and gas piping, scales can be treated mechanically or dissolved chemically. When mechanical removal procedures fail or are too costly, chemical dissolving is commonly used. For scales such as iron sulfides, they are known to be soluble in hydrochloric acid; hence, they can be used as a scale removal. However, when iron sulfide reacts with hydrochloric acid, it produces hydrogen sulfide, which is a very poisonous gas. Accordingly, it can be very toxic, and precautions must be taken [5]. Thus, an alternative chemical, such as chelating agents, can be used to prevent scale formation. Chelators have been proven as a more effective chemical technique in inhibiting and removing scales [6]. They are noted for their strong dissolving power, minimal corrosion, and good iron control, as well as some that can be degradable and environmentally friendly [7]. Likewise, they are known to be utilized in medical treatments to lower harmful heavy metal levels in the blood and tissues [8]. One of the proven chelators for treatment of iron sulfide scales is diethylenetriaminepentaacetic acid (DTPA) [9]. Hence, in this work, the focus is to explore green alternative solutions for inhibiting scale formation.

Currently, polyaspartic acid (PASP) and polyepoxysuccinic acid (PESA) are proven as effective scale inhibitors, and are widely used in the desalination industry, since they are environmentally friendly. A previous study showed that both achieved a corrosion inhibition efficiency of about 60%, which is relatively low; however, there is continuous research being conducted to improve their anti-corrosion properties by incorporating additional functional groups. However, adding zinc ions enhanced the efficiency, which reached 90% [10,11]. Moreover, in another study, PESA performance exceeded that of PASP in calcite and celestite control. Similarly, the anti-scaling performance of PESA was superior to that of PASP for calcite [12]. PESA’s effectiveness in scale control prompted the research group to try it on iron sulfide as a scale inhibitor. Moreover, several chemicals used for scale inhibition/removal have been developed, including formic acid, maleic acid, acetic acid, succinic acid, and citric acid [13]. Numerous studies also investigated several polymeric and co-polymeric oilfield scale inhibitors, including sulfonates and phosphonates [14]. Nevertheless, most of these compounds pollute the environment and are not environmentally friendly.

Molecular modeling tools have been extensively employed to investigate different scale inhibitors [13]. A tool such as density functional theory (DFT) has become a widely used method for understanding molecular electronic structures of a variety of metal complexes [15]. Hence, in this study, computational modeling will be used in observing the performance of PESA as a scale inhibitor. Fe’s structural and electronic characteristics with PESA complexes are investigated using DFT to better understand how the chelating agents bind to Fe at the molecular level. The formation of iron complexes is the key to the inhibition effect of PESA, as the formation of iron sulfide scales is hindered by sequestrating the free iron in the solution. 

Later, the geometry, binding energy, molecular orbital, and charge analysis of PESA were studied to understand its chemical interaction with iron sulfide scale. This would yield molecular views into how to present oil field chemicals that could be modified to provide more effective formulations. These formulations would be utilized to inhibit iron sulfide scale effectively.

Many research publications have investigated PESA as a green corrosion inhibitor; one paper utilized electrochemical, thermodynamic, and surface microscopic techniques, as well as computational approaches, to support the empirical findings of the inhibitory mechanism at the molecular level [10]. The performance of the PESA as a scale inhibitor was reported to be superior to PASP, and the anti-scaling properties of PESA were examined on cooling-water plant and oilfield tests, which are required to evaluate the effectiveness in the reservoir environment [16]. However, neither the Gaussian software nor any DFT computations were employed in these publications to investigate the electrochemical behavior or the properties of PESA structure, considering different forms.

**Figure 1:**
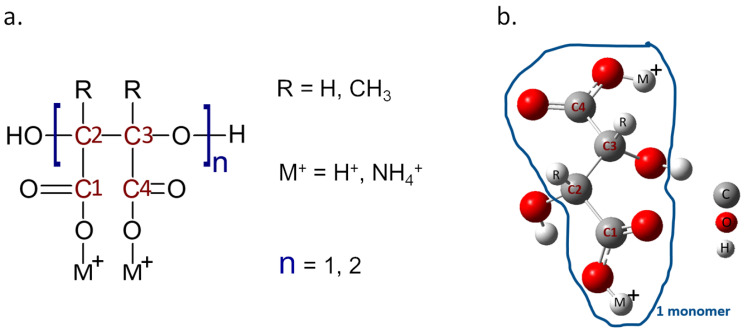
(**a**) Structure of PESA, highlighting the different R and M^+^ groups. (**b**) Model of the molecule as employed in the simulations.

The Gaussian 09 software was employed for the DFT calculations, to perform the molecular modeling that will assist in understanding the thermodynamic and kinetics of the chemical reactions during the scale deposition. While the characteristics of the oil wells are not explicitly included in the study, the interaction of PESA derived with iron is the first step to address the inhibition of scale formation in the wells themselves. The binding affinity is calculated between the designed molecules and the Fe. A total of eight compounds were developed and analyzed, as each molecule was considered in terms of monomer (n = 1) and dimer (n = 2) of PESA, and different forms of PESA were investigated by changing R and M groups (Figure 1). R represents two possible electron donating groups (H, CH_3_), while M^+^ represents two possible cation groups (H^+^ and NH_4_^+^), which turns the PESA compound into its acidic and its ammonia salt forms, respectively. Following that, the geometrical analysis findings are discussed and summarized in the results section. The analysis was supported by performing molecular orbital calculations to represent the behavior of the electrons in the molecules. Additionally, the HOMO-LUMO gaps and the electron affinity (EA) were calculated to determine whether it is feasible to excite the molecule or not, as well as to reveal whether PESA complexes generated with iron have strong reactivity, or whether the binding energies provide better stability for the complex. Finally, the charge analysis was carried out by utilizing the molecular electrostatic potential (ESP) map and Mulliken charge analysis. 

## 2. Computational Methods

Molecular modeling was performed in order to comprehend the thermodynamics and kinetics of chemical reactions during scale deposition. All DFT calculations were carried out using Gaussian 09 software at the B3LYP functional, with the 6–31 g (d) for non-metals, and lanl2dz for Fe. The solvation was employed by the polarizable continuum model (PCM) to represent water solvents. Figure 1 demonstrates the simulation visualization of one monomer of the PESA. The binding energy was calculated using the following equation:

(1) $$BE=E_{Fe-complex}-E_{compound}-E_{Fe}\;$$

## 3. Results and Discussion

### 3.1. Binding Energy and Geometrical Analysis

**Figure 2:**
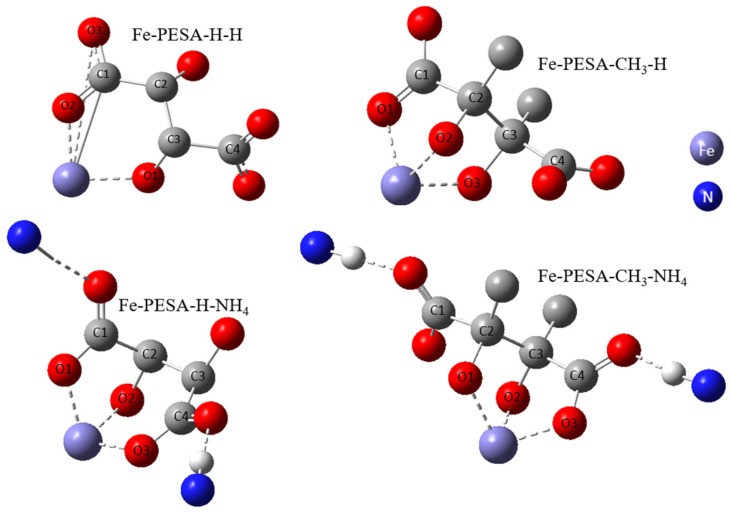
The optimized structure of Fe complex with 1-monomers of PESA molecule.

**Figure 3:**
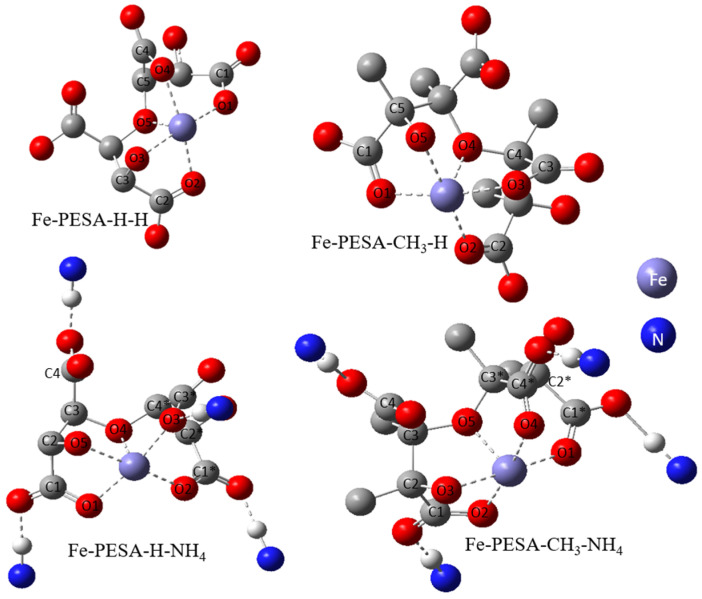
The optimized structure of Fe complex with 2-monomers of PESA molecule.

All DFT calculations were performed using the Gaussian software, as the DFT calculation was used to calculate the binding affinity between the designed molecules and the Fe using Equation (1). Various forms of PESA were considered by changing the R groups of C_2_ and C_3_, and considering H- and CH_3_- groups to study the effect of adding an electron-donating group in the binding affinity of PESA to the metal. Moreover, the standard acidic form of PESA (M^+^ = H^+^) and ammonium salt of PESA (M^+^ = NH_4_^+^) were studied to check the performance of PESA with different protonation. In total, eight molecules were considered and shown in the following form: Fe-PESA-R-M, referring to R and M^+^ groups. The molecules are Fe-PESA-H-H, Fe-PESA-CH_3_-H, Fe-PESA-H-NH_4_, and Fe-PESA-CH_3_-NH_4_; each was studied considering one monomer (1n) (Figure 2) and two monomers (2n) (Figure 3).

**Figure 4:**
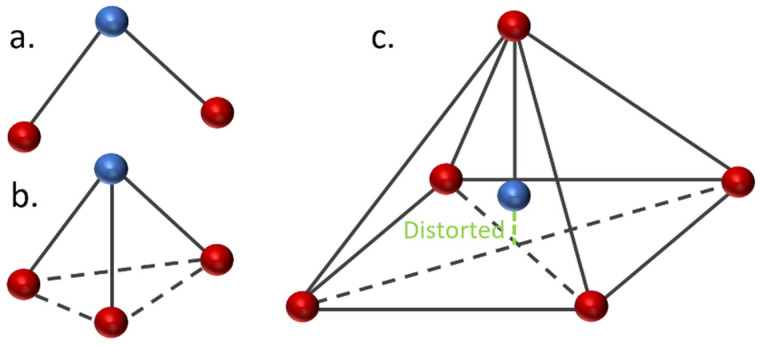
Demonstration of the geometrical structures of Fe-PESA-1n, (**a**) bent and (**b**) trigonal pyramidal, and Fe-PESA-2n (**c**), distorted square pyramidal.

The geometrical analysis findings are summarized in Table 1, clearly showing the higher coordination number for Fe-PESA-(2n) complexes compared to Fe-PESA-(1n) complexes. According to the optimized structures, Fe^II^ has two coordination numbers when binding to 1n of PESA-H-H complex, while the other 1n molecule has three coordination number-creating bent and trigonal pyramidal structures (Figure 4). On the other hand, the 2n Fe^II^-complexes have five coordination numbers forming a distorted square pyramidal structure, with the Fe located at the center of the geometrical structure, as demonstrated in Figure 4.

**Figure 5:**
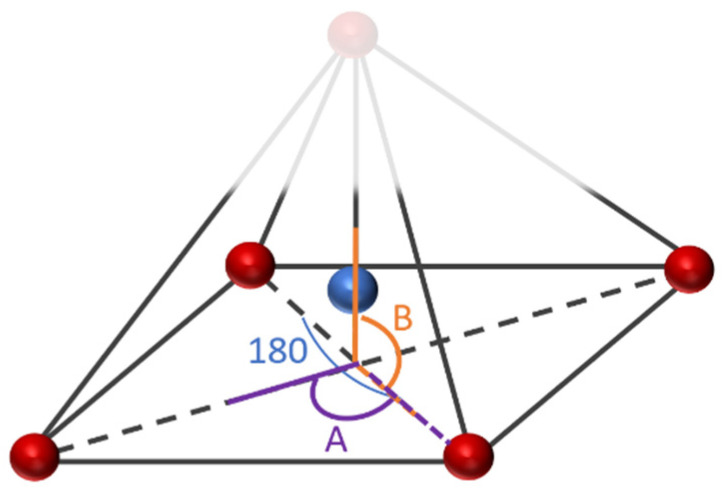
The studied side angles and (180) angle for the 2n Fe-PESA complexes in Table 1.

Table 1 summarizes all of the average distances between the Fe and the attached oxygen, as well as the average angles created around the Fe coordination (O-Fe-O) for all studied molecules. In addition, we focused on the coordination that complexed to Fe. The average H-O attraction bond length of the carboxylic groups that complexed with Fe^II^ were measured, as well as the H-N bond of the ammonium groups that were close to the same previously mentioned carboxylic groups. For 2n molecules, two types of angles were measured, the side angles of the square pyramidal structure and the angles close to 180°, as shown in Figure 5. The side angle is calculated from the average of A- and B- type angles shown in the figure. From the results in Table 1, all of the lengths of the O-Fe coordination bonds are similar to the values reported in the literature, which is ≈2 Å (Table 1). The shortest Fe-O bond was observed to be for the Fe-PESA-H-NH_4_ (1n) molecule, which had the highest binding energy (−319 kcal/mol). Similarly, other Fe-PESA molecules with NH_4_^+^ have the shortest Fe-O bond length and the highest binding energy. Furthermore, their O-H bonds are longest compared to other molecules without NH_4_. The angles of the 1n and 2n complexes for the bent, trigonal pyramidal, and distorted square pyramidal are ~90°, which is similar to the reported angles for these geometries. For PESA-1n molecules, the distortion in the geometrical structures is more pronounced with R- = CH_3_-, as they increase the steric hindrance and force the geometry to change.

The results of the calculated binding energies of all molecules are summarized in Table 2. The binding energy values are negative, indicating the good binding affinity of PESA molecules to Fe. In addition, it can be observed that 2n molecules had higher binding energy than the single monomer of PESA, due to the greater coordination number around Fe^II^. Furthermore, comparing the binding energy of the different PESA forms, Fe-H-NH_4_ showed higher binding energy than Fe-H-H, revealing that the negative carboxylate group in the ammonium salt of PESA has better binding energy. In general, the M^+^ (H, or NH_4_^+^) tends to detach from the oxygen atom when it binds to Fe, and then to the other oxygen away from Fe, in the same carboxylic group. This orientation forms a highly negatively charged oxygen with a higher affinity to Fe^II^. As the NH_4_^+^ molecules have the longest O-H bonds and the shortest Fe-O bonds, this reveals that they are more easily detached from the oxygen associated with Fe than the H^+^. Thus, all Fe-PESA with NH_4_^+^ has the highest binding energy compared to other molecules with H^+^ as an M^+^ cation. On the other hand, the addition of an electron-donating group, CH_3_-, increases the binding energy due to the increase in the overall negativity of PESA, thus increasing its ability to bind to the positive metal Fe^II^. For instance, the binding energy of Fe-H-H-1n is −275 kcal/mol, while Fe-CH_3_-H-1n has a higher binding energy of −320 kcal/mole. The effect of different groups and the resulting performance are further explained by the electronic structure in the charge and MO analyses.

In addition, the binding energy calculated for all molecules of PESA to Fe^II^ is close to other known chelating agents that have been shown to be efficient for scale inhibition/removal in the literature. For instance, the binding energy of PESA-H-H is −1151 kJ/mol, which is similar to the binding energy of DTPA to Fe^II^ which is −1135 kJ/mol [17]. Moreover, unlike DTPA, which was identified by the European Chemical Agency as a chemical that causes severe eye irritation [17], PESA is an environmentally friendly molecule. Thus, PESA has an acceptable binding affinity that is comparable to other known chelating agents, revealing its promising efficiency as a scale inhibitor/removal. Finally, the highest binding energy was observed for the Fe-CH_3_-NH_4_-2n molecule, since it includes both CH_3_ and NH_4_ groups with two monomers, and the overall binding energy order is H-H > CH_3_-H > H-NH_4_ > CH_3_-NH_4_.

### 3.2. Molecular Orbital

Molecular orbital calculations were carried out to describe the behavior of electrons in the molecules. According to the frontier orbital theory, the highest occupied molecular orbital (HOMO) and lowest unoccupied molecular orbital (LUMO) are helpful for measuring the chemical reactivity of the molecules [17]. A LUMO participates in the molecular interaction by taking in electrons, and the energy of the LUMO is related to the electron affinity (EA). In contrast, a HOMO participates in the molecular interaction as a donor, and has an energy related to the ionization potential (IP). Moreover, the HOMO-LUMO energy gap explains the charge transfer interactions within a molecule, which can be used to determine the basis of molecular electrical transport [18]. Thus, through HOMO and LUMO values, the electron affinity (EA) and HOMO-LUMO gap were calculated for all complexes with 1n and 2n. When the gap is small, the complex is characterized by a high chemical reactivity [19], whereas when it is larger, it shows that the molecule forms with higher thermodynamic stability [18].

**Figure 6:**
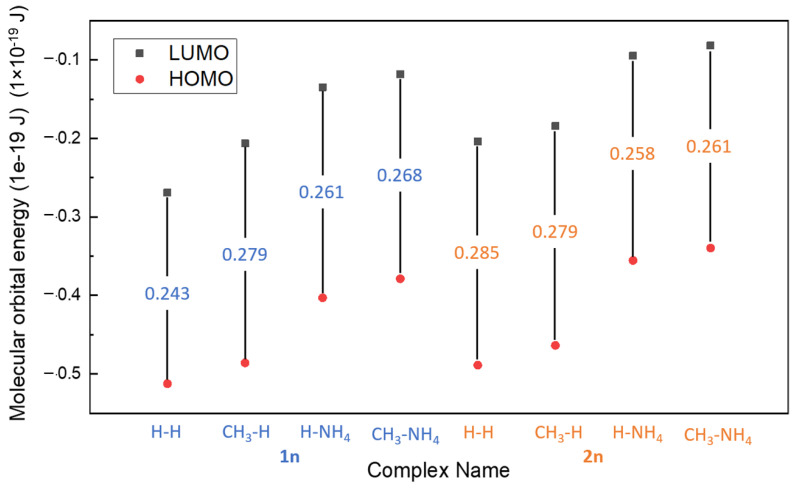
The HOMO-LUMO gap of different complexes.

The calculated HOMO-LUMO gap and electron affinity (EA) are summarized and demonstrated in Table 3 and Figure 6. The gaps obtained for all molecules range from 0.3834 to 0.418 × 10^−19^ J, and the HOMO-LUMO gap decreases for all molecules as they bind to Fe^II^, as shown in Table 3 (range from 0.243 to 0.285 × 10^−19^ J). Comparing the results of the previous study, DTPA with Fe^II^ ion, which was proven as an effective iron sulfide scale removal, acquired a HOMO-LUMO gap of 8.861 × 10^−19^ J, which is high when compared to PESA complexes with a single Fe (<0.418 × 10^−19^ J) [17]. The lower the gap of the PESA-Fe complexes means that this complex could be less stable thermodynamically and more reactive than DTPA-Fe. Good reactivity of PESA complexes formed with iron was observed from the results, as the HOMO-LUMO gaps were small. However, the standard PESA developed the largest HOMO-LUMO gap, which signifies better stability. Although the gaps of PESA complexes are small, their calculated binding energy is still comparable to DTPA (−1135 kJ/mol).

The order of the gap for Fe-PESA-2n complexes is as follows: H-H > CH_3_-H > H-NH_4_ > CH_3_-NH_4_ (Table 3), signifying that CH_3_-NH_4_ has the smallest value; however, it has the highest binding energy, as discussed previously. The results may convey that CH_3_-NH_4_ can be a better chelating agent, as it reacts with the Fe to form a stable complex, which is shown from the EA results [20]. Furthermore, it is shown in Figure 6 that CH_3_-NH_4_ has a lower HOMO; hence, it tends to be more stable. An interesting conclusion is that the gap between HOMO and LUMO is lowered when the PESA complex with Fe^II^ implies good inhibition efficiency [19] (Table 3). Furthermore, electron affinity (EA) was calculated by Equation (2),

(2) EA=−ELUMO

which refers to the amount of energy released when an electron is added to a neutral atom to form a negatively charged ion. Values were positive for all the molecules, denoting that the molecules accept more electrons, indicating stability. All complexes exhibited this behavior, as their LUMO values were negative. The CH_3_-NH_4_ molecule has the lowest value of LUMO (0.118), while H-H has the largest positive value (0.269).

**Figure 7:**
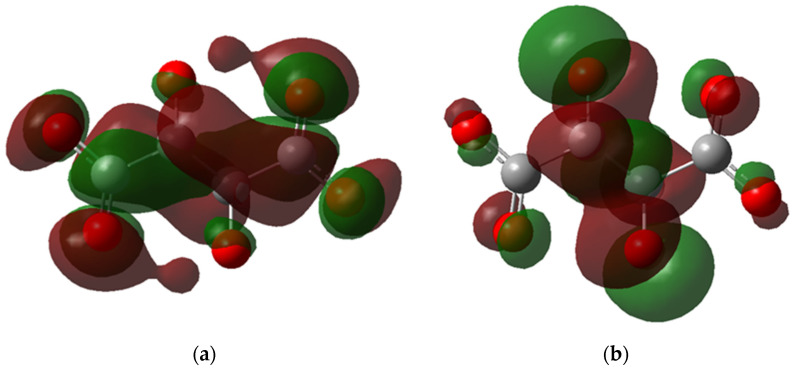
The molecular orbital maps, (**a**) LUMO and (**b**) HOMO, of the PESA-H-H-1n molecule.

**Figure 8:**
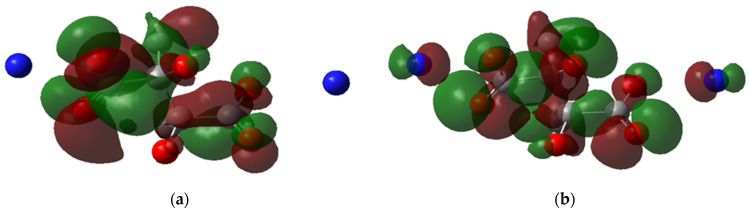
The molecular orbital maps, (**a**) LUMO and (**b**) HOMO, of the PESA- CH_3_-NH_4_-1n molecule.

Another observation from the HOMO and LUMO maps is the delocalization of electrons. In Figure 7a, the LUMO of H-H is delocalized in the carboxylic group and the central carbon atoms; however, the HOMO is localized around the oxygens of the carboxylic groups (Figure 7b), reflecting that the electrons are present in the negative side of the carboxylic group where the binding to Fe^II^ occurs. Meanwhile, for CH_3_-NH_4_, the HOMO is delocalized around the nitrogen atoms and carboxylic groups (Figure 8a), indicating that the ammonium cations indirectly affect the ligand complexation reaction. This observation could explain why the ammonium salt of PESA performs better than standard PESA. The reverse is observed for LUMO, where the delocalization can be dominant only in the carboxylic groups (Figure 8b). 

### 3.3. Charge Analysis

**Figure 9:**
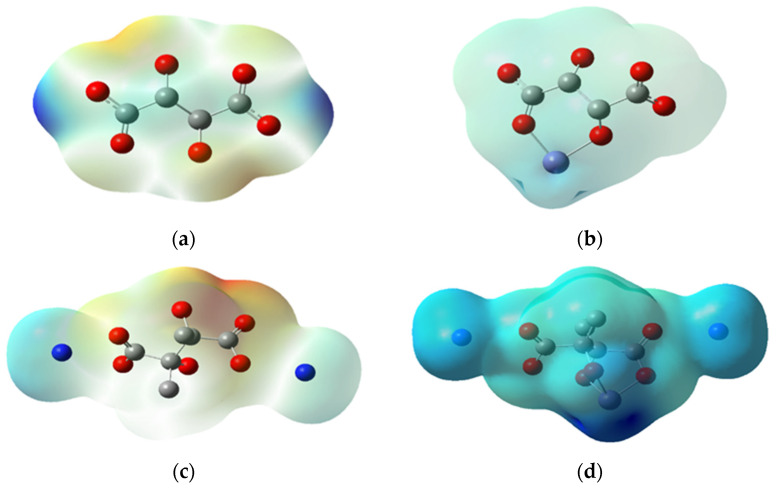
Electrostatic potential for 1n of (**a**) H-H (0.0737 eV), (**b**) Fe-H-H (0.9110 eV), (**c**) CH_3_-NH_4_ (0.1190 eV) and (**d**) Fe-CH_3_-NH_4_ (0.4240 eV).

The charge analysis was performed using the molecular ESP map, which depicts the positive and negative charged regions of a molecule. From negative to positive, the ESP increases from red to orange to yellow to green to blue, going from the high negative region to the positive region [17] as indicated in Figure 9. The overall ESP of the complexes denotes that it is electronegative; thus, it is expected to attract a positively charged Fe. Moreover, it clearly shows that when iron complexes with PESA, the charge becomes positive, which is more pronounced than ammonium salt of PESA (Figure 9b,d respectively). The Mulliken charges were used to analyze the distribution of electronic charges within the molecules. The results in Table 4 show that the order of the Fe positive charges for 2n is as follows: H-H > CH_3_-H > H-NH_4_ > CH_3_-NH_4_. The positivity of Fe decreases with higher binding energy due to greater charge transfer. Moreover, the complexes with the electron donating group and PESA in the ammonium salt form have oxygens with greater negative charges; consequently, CH_3_-NH_4_ complexes have the highest negative charges on oxygens.

In normal cases, the negativity of the oxygen atoms decreases after coordinating with metals as they donate the electrons; however, an interesting observation in our case is that for all molecules, the oxygen atoms’ negativity increases after they complex with Fe^II^ (Table 5). The reason behind this is the dissociation of the M^+^ cation from the carboxylic group for the standard acidic PESA and its ammonium salt, leaving the oxygens with a higher negative charge. Thus, in all forms of PESA, the carboxylic group tend to lose the hydrogen to complex with the Fe^II^. However, for the ammonium salt of PESA, the negativity of oxygen is increased more significantly compared to the standard PESA complexes, due to the transfer of the negative charges from nitrogen atoms to the complex, as shown in the charge analysis (Table 5). For instance, the charge of N in CH_3_-NH_4_ for 1n is −0.624, which decreases to −0.317 after PESA complexes to Fe^II^. Hence, the ammonium salts of PESA showed better performance than the standard acidic form.

## 4. Conclusions

The electrochemical and geometrical structure of PESA as a scale inhibitor was investigated using the Density Functional Theory (DFT) to understand the kinetics and thermodynamics of the chemical reactions during scale inhibition. Different forms of PESA were considered for the addition of an electron donating group (CH_3_-), as an R group was studied, and the ammonium cation was considered to be M^+^ instead of H^+^. The scale is represented as Fe^II^ ions, and PESA is modeled as (n = 1, and 2). The binding affinity(BE) was calculated for all eight PESA molecules, and the geometrical structure was investigated. The 1n molecules had bent and trigonal pyramidal structures, while the 2n molecules had a greater coordination number (5), creating a distorted square pyramidal structure. The distortion was more pronounced with the CH_3_- groups due to the steric hindrance. In addition, the calculated binding energy for all molecules is comparable to the affinity of well-known chelating agents, such as DTPA. Furthermore, the highest binding energy was observed for the ammonium salt of PESA with the electron donating group.

The HOMO-LUMO gap, molecular orbital localization, electron affinity, and charge analysis were used to explain the binding energy results and give more details about the molecules’ stability and chemical reactivity. First, the values of the HOMO-LUMO gap for all molecules were small compared to the available chelating agents, revealing the high reactivity of PESA complexes. In addition, the low values of their HOMOs reflect the high inhibition performance of PESA, and the value was the lowest (highest negative value) for CH_3_-NH_4_ complexes. Furthermore, the localization of the HOMO orbital of CH_3_-NH_4_ complexes reveals the participation of the NH_4_^+^ group in the complexation. The charge analysis shows the same conclusion, as the complexes with CH_3_- group and the ammonium salt of PESA have the highest levels of negative oxygen atoms. Moreover, the charge analysis reveals the charge transfer that occurs from the ammonium cation, increasing the negativity of PESA, and, thus, the binding affinity as well. Finally, the EA results for all molecules were positive, revealing their stability. PESA complexes have high potential for improving the scale inhibitor mechanism, due to their strong bonding with iron. 

## Figures and Tables

**Table 1:** Geometrical analysis of the optimized molecular structure of Fe-PESA complexes, including the distances and angles.

Molecules	Distances			Angles		Geometry
	Fe-O	O-H	N-H	O-Fe-O		
1n						
Fe-PESA-H-H	1.95	0.97	-	90.6		Bent
Fe-PESA-CH_3_-H	1.98	0.98	-	83.0		Trigonal pyramidal
Fe-PESA-H-NH_4_	1.93	1.65	1.03	90.3		
Fe-PESA-CH_3_-NH_4_	1.94	1.67	1.03	85.3		
2n						
				Side angle	180° angle	
Fe-PESA-H-H	2.02	0.98	-	84.2	166.7	Distorted Square Pyramidal
Fe-PESA-CH_3_-H	2.03	0.98	-	87.94	170.99	
Fe-PESA-H-NH_4_	1.99	1.60	1.08	86.65	171.82	
Fe-PESA-CH_3_-NH_4_	1.97	1.58	1.03	88.99	174.31	

**Table 2:** The energies of the optimized molecules with the calculated binding energy, according to Equation (1). The energy of Fe^II^ is −1262.9 Hartree.

	PESA	Fe-PESA	Binding Energy
	Hartree		kcal/mol
1n			
Fe-PESA-H-H	−607.5680	−1870.937	−275
Fe-PESA-CH_3_-H	−686.168	−1949.566	−294
Fe-PESA-H-NH_4_	−720.726	−1984.166	−320
Fe-PESA-CH_3_-NH_4_	−799.323	−2062.760	−318
2n			
Fe-PESA-H-H	−1138.665	−2402.059	−291
Fe-PESA-CH_3_-H	−1295.846	−2559.275	−313
Fe-PESA-H-NH_4_	−1364.976	−2628.457	−346
Fe-PESA-CH_3_-NH_4_	−1522.150	−2785.663	−366

**Table 3:** The HOMO-LUMO gap of the optimized polymers and their corresponding Fe-complex, and the electron affinity (EA) values for the Fe-PESA complexes only. The energy values are in 1 × 10^−19^ J.

Molecules	PESA			Fe-PESA			
	LUMO	HOMO	Gap	LUMO	HOMO	Gap	EA
1n							
H-H	−0.058	−0.466	0.408	−0.269	−0.513	0.243	0.269
CH_3_-H	−0.048	−0.463	0.415	−0.206	−0.486	0.279	0.206
H-NH_4_	−0.007	−0.421	0.413	−0.135	−0.403	0.268	0.135
CH_3_-NH_4_	−0.014	−0.433	0.418	−0.118	−0.379	0.261	0.118
2n							
H-H	−0.073	−0.458	0.385	−0.204	−0.489	0.285	0.204
CH_3_-H	−0.066	−0.449	0.383	−0.184	−0.464	0.279	0.184
H-NH_4_	−0.035	−0.421	0.387	−0.095	−0.355	0.261	0.095
CH_3_-NH_4_	−0.027	−0.411	0.384	−0.082	−0`.340	0.258	0.082

**Table 4:** The Mulliken charges of the oxygen atoms coordinated with Fe.

Complex	Oxygen		Fe	Complex	Oxygen		Fe
	Before	After			Before	After	
1n				2n			
H-H	−0.388	−0.438	1.457	H-H	−0.252	−0.333	0.885
	−0.390	−0.368			−0.256	−0.338	
CH_3_-H	−0.375	−0.375	1.219		−0.364	−0.383	
	−0.367	−0.343			−0.329	−0.265	
	−0.324	−0.297			−0.270	−0.306	
H-NH_4_	−0.409	−0.402	1.051	CH_3_-H	−0.258	−0.304	0.787
	−0.393	−0.385			−0.25	−0.294	
	−0.486	−0.395			−0.353	−0.298	
CH_3_-NH_4_	−0.377	−0.514	1.056		−0.321	−0.264	
	−0.369	−0.399			−0.332	−0.302	
	−0.394	−0.390		H-NH_4_	−0.266	−0.303	0.719
					−0.283	−0.47	
					−0.378	−0.362	
					−0.379	−0.385	
					−0.375	−0.393	
				CH_3_-NH_4_	−0.268	−0.31	0.674
					−0.276	−0.36	
					−0.365	−0.336	
					−0.408	−0.44	

**Table 5:** The Mulliken charges of the M^+^ and H^+^ of the carboxylic groups of all compounds.

Complex	Before				After			
	O-M (H^+^/NH_4_^+^)				O-M (H^+^/NH_4_^+^)			
	O	H*	N	H	O	H*	N	H
1n								
H-H	−0.257	0.325	-	-	−0.278	0.348	-	-
	−0.257	0.325	-	-	−0.204	0.350	-	-
CH_3_-H	−0.375	0.306	-	-	−0.375	0.391	-	-
	−0.367	0.309	-	-	−0.343	0.366	-	-
	−0.271	0.321	-	-	−0.184	0.360	-	-
	−0.278	0.331	-	-	−0.223	0.342	-	-
H-NH_4_	−0.409	0.286	-	-	−0.402	0.357	-	-
	−0.427	0.296	-	-	−0.383	0.304	-	-
	−0.532	0.280	−0.338	0.298	−0.497	0.286	−0.317	0.309
	−0.304	0.305	−0.626	0.249	−0.446	0.278	−0.315	0.309
CH_3_-NH_4_	−0.377	0.299	-	-	−0.514	0.310	-	-
	−0.394	0.299	-	-	−0.390	0.367	-	-
	−0.290	0.307	−0.624	0.249	−0.451	0.276	−0.317	0.307
	−0.336	0.295	−0.585	0.258	−0.423	0.286	−0.312	0.312
2n								
H-H	−0.256	0.327	-	-	−0.338	0.393	-	-
	−0.288	0.327	-	-	−0.234	0.349	-	-
	−0.260	0.328	-	-	−0.191	0.360	-	-
	−0.270	0.323	-	-	−0.306	0.390	-	-
CH_3_-H	−0.250	0.326	-	-	−0.294	0.394	-	-
	−0.275	0.321	-	-	−0.186	0.358	-	-
	−0.259	0.317	-	-	−0.198	0.347	-	-
	−0.250	0.325	-	-	−0.183	0.349	-	-
	−0.353	0.296	-	-	−0.298	0.364	-	-
	−0.346	0.297	-	-	−0.302	0.315	-	-
H-NH_4_	−0.283	0.299	−0.606	0.254	−0.470	0.314	−0.326	0.308
	−0.319	0.308	−0.617	0.250	−0.457	0.277	−0.329	0.302
	−0.296	0.296	−0.596	0.256	−0.463	0.277	−0.324	0.305
	−0.324	0.318	−0.617	0.252	−0.462	0.272	−0.327	0.303
CH_3_-NH_4_	−0.293	0.309	−0.624	0.248	−0.456	0.276	−0.324	0.304
	−0.276	0.302	−0.615	0.269	−0.477	0.280	−0.323	0.305
	−0.302	0.309	−0.619	0.247	−0.450	0.273	−0.331	0.301
	−0.280	0.301	−0.607	0.254	−0.438	0.276	−0.329	0.301
	−0.365	0.285	-	-	−0.336	0.319	-	-
	−0.372	0.288	-	-	−0.332	0.287	-	-

Note: The hydrogens of NH_4_^+^ are denoted as H* for the hydrogen connected to O, and the average of the other hydrogens is denoted as H in the table.

## Data Availability

No Data available.
